# Soil and Leaf Ionome Heterogeneity in *Xylella fastidiosa* Subsp. *Pauca*-Infected, Non-Infected and Treated Olive Groves in Apulia, Italy

**DOI:** 10.3390/plants9060760

**Published:** 2020-06-17

**Authors:** Laura Del Coco, Danilo Migoni, Chiara Roberta Girelli, Federica Angilè, Marco Scortichini, Francesco Paolo Fanizzi

**Affiliations:** 1 Department of Biological and Environmental Sciences and Technologies, University of Salento, Prov.le Lecce-Monteroni, I-73100 Lecce, Italy; laura.delcoco@unisalento.it (L.D.C.); danilo.migoni@unisalento.it (D.M.); chiara.girelli@unisalento.it (C.R.G.); federica.angile@unisalento.it (F.A.); 2 Council for Agricultural Research and Economics-Research Centre for Olive, Fruit Trees and Citrus Crops, Via di Fioranello, 52, I-00134 Roma, Italy; marco.scortichini@crea.gov.it; 3 University of Salento Local Unit of Consorzio Interuniversitario di Ricerca in Chimica dei Metalli nei Sistemi Biologici (CIRCMSB), Via Celso Ulpiani, 27-70126 Bari, Italy

**Keywords:** olive quick decline syndrome, inductively coupled plasma atomic emission spectroscopy analysis, macronutrients, micronutrients, epidemiology, pathogen control

## Abstract

*Xylella fastidiosa* subsp. *pauca* is responsible for the “olive quick decline syndrome” (OQDS) in Salento (Apulia). The main epidemiological aspects of the syndrome are related to the pathogen spread and survival in the area, and to the biology of the insect vector. The assessment of the macro and microelements content (i.e., ionome) in soil and leaves could provide basic and useful information. Indeed, knowledge of host ionomic composition and the possibility of its modification could represent a potential tool for the management of diseases caused by *X. fastidiosa*. Therefore, soil and leaf ionomes of naturally infected, not infected, and zinc–copper–citric acid biocomplex treated trees of different areas of Apulia and the bordering Basilicata regions were compared. We observed that soil and leaf ionomic composition of olive farms growing in the pathogen-free areas north of the Salento Barletta-Andria-Trani BAT (Apulia) and Potenza PZ (Basilicata, Apulia bordering region) provinces is significantly different from that shown by the infected olive groves of the Salento areas (LE, BR, TA provinces). In particular, a higher content of zinc and copper both in soil and leaves was found in the studied northern areas in comparison to the southern areas. This finding could partly explain the absence of OQDS in those areas. In the infected Salento areas, the leaf ionomic profile resulted as being markedly different for the biocomplex treated compared to the untreated trees. A higher zinc content in leaves characterized treated with respect to untreated trees. On the other hand, among the not-infected trees, *Xylella*-resistant Leccino showed higher manganese content when compared with the higher pathogen sensitive Ogliarola salentina and Cellina di Nardò. According to these results, soil and olive leaf ionome could provide basic information for the epidemiologic study and possible control of *X. f.* subsp. *pauca* in Apulia.

## 1. Introduction

*Xylella fastidiosa* is a plant pathogenic bacterium that causes damage to many crops, mainly in North, Central and South America. Recently, this quarantine phytopathogen has enlarged its distribution by reaching several European countries and infecting both cultivated and wild plants [1]. *X. f.* subsp. *pauca* is responsible for the “olive quick decline syndrome” (OQDS) in the Salento area (Apulia region, southern Italy) [2]. Currently, it is estimated that about 6,500,000 trees are infected by this bacterium [3]. The main symptoms are leaf, twig, and branch wilting, followed by the death of the plant. The pathogen was, most probably, introduced in the area from Central America through the circulation of infected Coffee plants [4,5]. So far, a single clonal lineage of this subspecies, namely the sequence type 53, is associated with OQDS in all infected areas of Salento [6,7]. According to the European quarantine legislation, three areas have been established for a better management of the disease: (1) the “infected” area where the pathogen is spread and the eradication measures are not feasible (i.e., the southernmost area of the Salento peninsula); (2) the “containment” area that borders the “infected” one and where infected olive trees must be uprooted; (3) the “buffer” area where, within a radius of 100 m [8,9] that starts from the infected tree, all olive trees must be uprooted. Debatably, in future, the length of this radius could be reduced [10]. The last two areas are surveyed and monitored by the regional phytosanitary service for assessing the occurrence of the pathogen [11]. The area north of the “buffer” is retained and declared “free” from the pathogen upon the surveys and laboratory analyses performed by ad hoc Institutions of Apulia Region.

Concerning the epidemiology of the disease, some studies have established that, in Apulia, the pathogen can survive and infect some plant species other than the olive [2]. Moreover, the meadow spittlebug *Philaenus spumarius* is considered the main insect vector enabling the spread of the pathogen within and between the olive groves [12]. Data on the expansion of the disease indicate that, at the time of the first report of October 2013, the OQDS was already established on about 10,000 ha in the Gallipoli area (Lecce province) [13]. The vector also spreads the pathogen in the affected area at a speed of 20 km per year [14], with the possibility of a higher spread rate, due to the occurrence in the area of non-olive hosts [15]. In addition, the particular topology of Apulia olive groves, regularly extending over hundreds of kilometers, makes the possibility that *X. f*. subsp. *pauca* will persist in the territory very high [16].

Other aspects of OQDS epidemiology could be related to the nutrients content in the soil and leaf. The analysis of the complete profile of the mineral nutrients and trace elements (i.e., the ionome *sensu*) [17] can also contribute to assessing the physiological state of the plant in relationships with the pathogen infection. Some studies, indeed, clearly indicated a correlation between the content of some ions into the leaf and the virulence of *X. fastidiosa* [18]. In *Nicotiana tabacum,* the ionome change correlates with the virulence of isolated *X. fastidiosa* and according to the different subspecies of the pathogen [19]. In addition, zinc detoxification in the host plant is required for triggering the full virulence of the pathogen [20]. Besides micronutrients, some other ions, such as calcium, are also involved in the virulence mechanisms of *X. fastidiosa* by favoring the adhesion of the pathogen cell to the xylem vessels, the biofilm formation and the twitching motility of the bacterium [21]. Concerning *X. f*. subsp. *pauca*, the highest manganese leaf content found into the olive cultivar Leccino could be related to its lower susceptibility to OQDS [22]. On the other hand, the same micronutrient, when supplemented to the standard media, caused an increase in biofilm formation and in cell-to-cell attachment of *X. f*. subsp. *fastidiosa* [18]. Previously, we showed that in the area where the OQDS outbreaks were firstly noticed (i.e., the Gallipoli area) there is a general low content of molybdenum and manganese in the soil and a low bioavailability of copper and molybdenum in the leaf of *X. f*. subsp. *pauca*-infected olive trees [23]. An accumulation of calcium and magnesium was also observed in *Vitis vinifera* leaves infected by *X. f.* subsp. *fastidiosa* [24]. Moreover, it was suggested that the knowledge of host ionomic composition and the possibility of its modification represent a potential tool for the management of the diseases caused by *X. fastidiosa* [20]. Within this context, we observed that the supply of a zinc–copper–citric acid biocomplex to the canopy of *X. fastidiosa* subsp. *pauca*-infected olive trees during spring and summer allowed to reduce both the field symptoms (i.e., leaf and twig die-backs) and pathogen cell densities within the leaves [25] as well as a rapid re-programming of the basic metabolism of the infected tree [26,27].

In order to start to verify whether differences in soil and leaf ionome composition between olive groves of the “infected” and “free” areas of Apulia are consistent, we have assessed the micronutrients content together with calcium, sodium, and magnesium in the soil and leaves of olive groves in different areas of Apulia and bordering region Basilicata. Beside the infected Salento area (Lecce LE, Brindisi BR and Taranto TA provinces) we have also included olive trees growing outside the areas where the *X. f.* subsp. *pauca* has been reported in the analyses. These occur more north than Salento and include the provinces of Barletta-Andria-Trani (BAT) (Apulia) and Potenza (PZ) (outside Apulia in the bordering region Basilicata). A full comparison has been performed for the areas characterized by the presence or absence of the pathogen. In addition, to assess the variation in the leaf ionome composition of olive trees that have received the biocomplex in recent years, we have also compared trees that received spray treatments with the untreated infected trees. We have observed that the soil and leaf ionomic composition of olive farms growing in north Salento areas is significantly different from that shown by the olive groves in the infected areas. We have also found that the biocomplex-treated and untreated olive trees of the infected areas have markedly different leaf ionomic profiles. Ions such as zinc, copper and sodium would seem somehow linked to the OQDS.

## 2. Results

### 2.1. Soil Analyses

**Figure 1:**
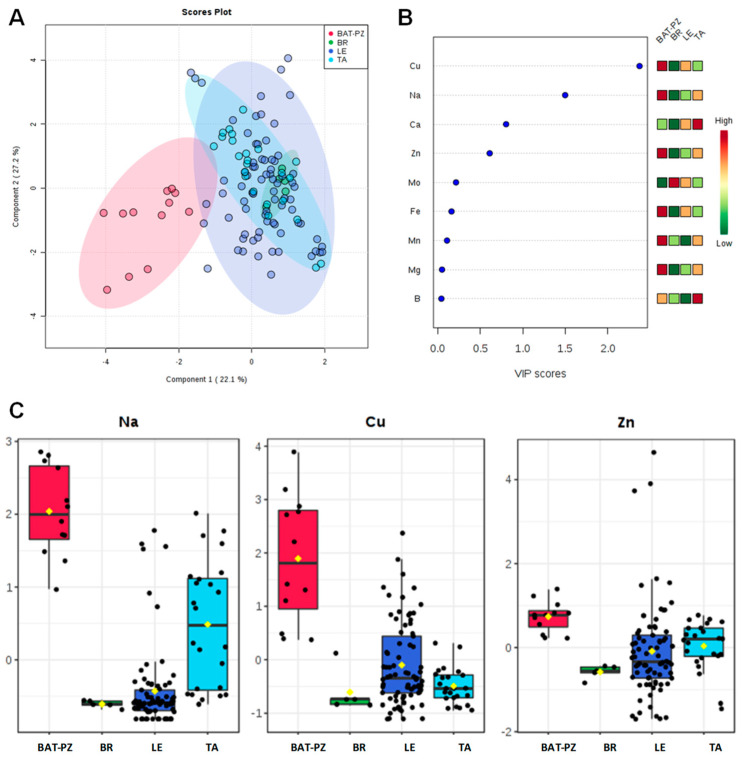
PLS-DA scoreplot (**A**), importance of variables ranked by VIP (Variable Importance in Projection score) (**B**) and box plot for the discriminant elements (**C**) obtained for soil samples, with a VIP score > 1. (Figure 1B: the colored boxes on the right indicate the relative increase/decrease of the corresponding variable in each group). BAT: Barletta-Andria-Trani; LE, BR, TA: Lecce, Brindisi, Taranto provinces (Apulia); PZ Potenza province (Basilicata, Apulia bordering region).

Mean and standard error values for the macro (Ca, Na, Mg) and micro (B, Cu, Fe, Mn, Mo, Zn) elements content in soils are shown in Table 1. In order to highlight significant differences in their amount, the Tukey Honestly Significant Difference (HSD) test for multiple comparisons of groups was also applied (Table 1 and Table S1 in SI). According to the indicated normal mean value content in soil of each element assessed [28,29,30,31,32,33,34,35], relevant differences among the ionomic profiles in soils of Apulia provinces have been observed. Olive groves located in the Barletta-Andria-Trani (BAT) and Potenza (PZ) provinces, north of the infected areas, showed a significantly higher content of Cu, Zn and Mn when compared to the other provinces. BAT and PZ olive groves also showed a significantly higher content of Na close to the limit for sodic soils (i.e., 3850.00 ± 243.27 mg kg^−1^) [29] (Figure S1 and Table S2 in SI). On the other hand, the LE province olive groves showed the lowest Mn content, whereas those of BR showed, in addition to a low Cu and Zn content, a significant low level of Mg. Olive farms located in TA province showed a mean Cu content close to the low limit for the normality [36]. A further level of investigation was performed by calculating the correlation matrix based on Pearson’s coefficient for all the measured elements (Table 2). A general overview on the potential linear relationship among the metals macronutrients and micronutrients was obtained. A high level of correlation was found for 14 couple of elements, namely B/Fe, B/Ca, B/Mg, B/Mn, B/Zn, Ca/Fe, Ca/Mg, Ca/Mn, Cu/Zn, Fe/Mg, Fe/Mn, Fe/Zn, Mg/Zn, and Mn/Zn, with significance at *p* < 0.001 (Table 3). It should be also noted that, in some cases, significant negative correlation values (i.e., B/Ca, Ca/Cu, Ca/Fe, Ca/Mn) were observed (Table 2 and Figure S2 in SI). A confirmation about the different ionomic composition of the BAT and PZ soils in comparison with those of Salento (LE, BR, TA) has been provided by the PLS-DA model (accuracy = 0.80, R^2^ = 0.43, Q^2^ = 0.33). This model clearly differentiated the soils of northern areas from the ones sampled in the Brindisi, Taranto and Lecce provinces (Figure 1A). In particular, as shown by the VIP scores (Figure 1B,C) and detailed in the histograms for the two main discriminant metabolites (with a VIP score > 1), Na and Cu were the main variables responsible for the discrimination among the four groups. Soil pH was always higher than 7.5, and even higher than 8.0 for the BAT, PZ and TA samples (Table 1).

### 2.2. Leaf Analyses

**Figure 2:**
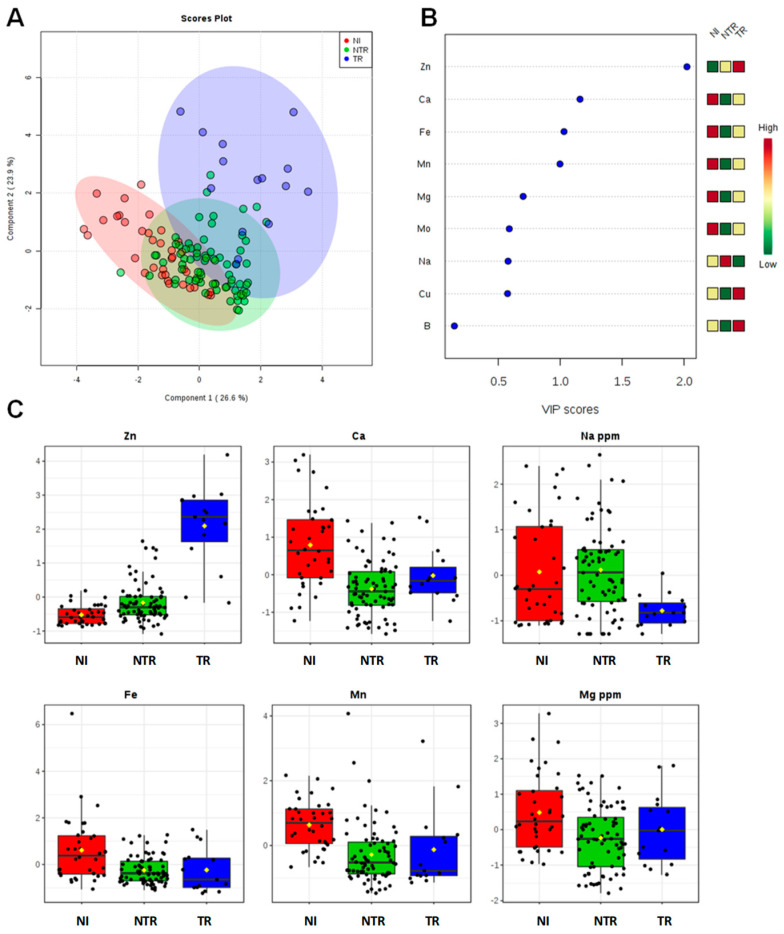
PLS-DA scoreplot (**A**), importance of variables ranked by VIP (Variable Importance in Projection score) (**B**) and box plot for the discriminant elements (**C**) obtained for olive leaf samples. (Figure 2B: the colored boxes on the right indicate the relative increase/decrease of the corresponding variable in each group). TR (farm treated with zinc–copper–citric acid), NTR (farm not treated and infected by *Xylella fastidiosa* subsp. *pauca*) and NI (farm not infected by *Xylella fastidiosa* subsp. *pauca*).

**Figure 3:**
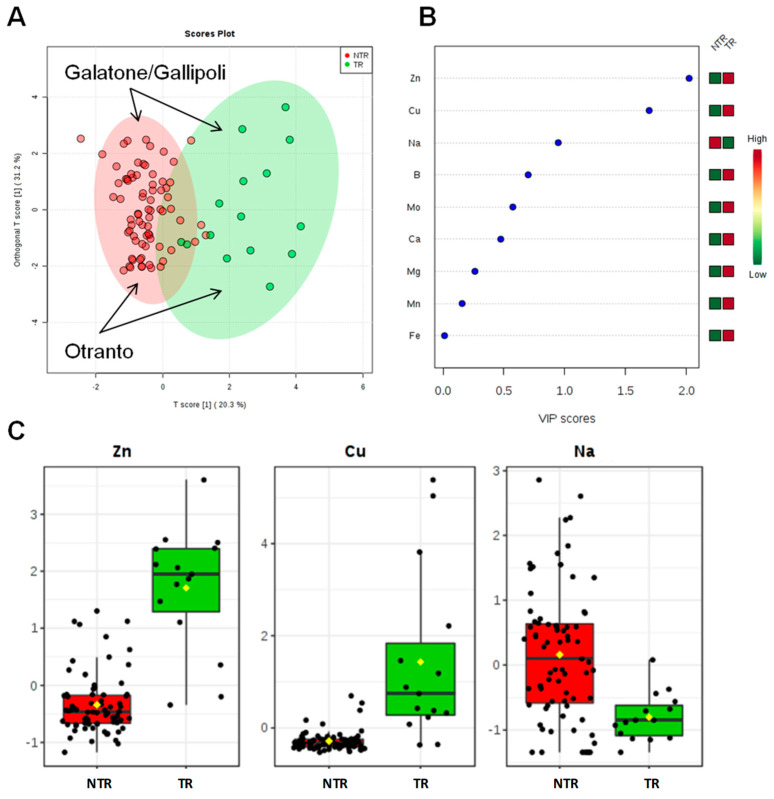
OPLS-DA scoreplot (**A**), importance of variables ranked by VIP (Variable Importance in Projection score) (**B**) and box plot for the discriminant elements (**C**) obtained for olive leaf samples for treated (TR) and not treated (NTR) infected olive leaves.

The ionomic profile of leaf samples was determined by ICP-AES analysis of the olive trees leaves located in the different municipalities (Table 3). For all the measured elements, univariate and multivariate statistical analyses (PCA, PLS-DA, OPLS-DA) were performed. The first approach is based on a multivariate statistical analysis model, performed using the whole data for the Dentamet^®^-treated (TR), not treated-infected (NTR), and not infected (NI) olive leaf samples. Both the PCA (Figure S3 in SI) and the PLS-DA (accuracy = 0.75, R^2^ = 0.61, Q^2^ = 0.55, Figure 2) models showed a clear separation of TR from the NI and NTR olive leaf samples, mainly due to a higher content of Zn in the treated trees olive leaves. The most important variables identified by the PLS-DA model ranked by VIP (Variable Importance in Projection score) are shown in Figure 2B and One-way ANOVA with post hoc analysis (Table S3 in SI) for multiple comparisons of groups (TR, NTR, NI) indicate significant variables with *p* < 0.05. Beside Zn, a higher relative content of Ca and Mg and a low relative content of Na were also found in NI olive leaf samples, whereas NTR leaf showed a relative higher content of Na. To note that leaves of TR trees showed the lowest Na content (Figure 2C). Furthermore, both unsupervised (PCA) and supervised (OPLS-DA) models were built for Dentamet^®^-treated (TR) and not treated-infected (NTR) olive leaf samples. Despite the different number of samples per cluster, a robust PLS-DA (accuracy = 0.95, R^2^ = 0.71, Q^2^ = 0.61, data not shown) and OPLS-DA (R^2^X = 0.51, R^2^Y = 0.64, Q^2^ = 0.60) models were obtained. The OPLS-DA scoreplot (Figure 3A) shows a clear separation between the two groups (NTR and TR). It should be noted that, in this case, all the analyzed samples originate from different municipalities among the Lecce province and refer to infected olive trees. Differences in micro/macronutrient content were also analyzed by the VIP score (Figure 3B), in which a marked variation in Zn, Cu and Na content (VIP score > 1) was observed between the two groups of samples (NTR and TR). In particular, by analyzing the detailed concentration of variables per group, a high relative content of Zn and Cu and a low relative content of Na were observed for TR with respect to NTR leaf samples (Figure 3C). Moreover, the NTR leaf samples showed a very low Cu content (Figure 3C). Interestingly, the same type of intra-class variation was observed in the two OPLS-DA groups (NTR and TR). The samples from Galatone/Gallipoli municipalities lie in the upper part, whereas those from the Otranto district and its surrounding municipalities are positioned in the lower side of the OPLSDA scoreplot (Figure 3A).

**Figure 4:**
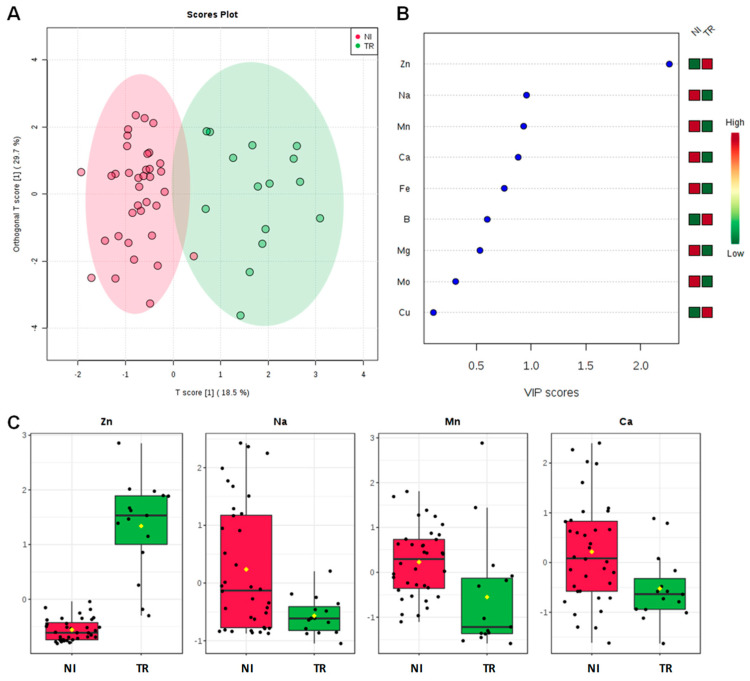
OPLS-DA scoreplot (**A**), importance of variables ranked by VIP (Variable Importance in Projection score) (**B**) and relative box plot for the discriminant elements (**C**) for TR (from Cellina di Nardò and Ogliarola salentina, all Lecce districts) and not infected samples (NI) olive leaves from Brindisi, Taranto and Barletta-Andria-Trani and Potenza provinces.

**Figure 5:**
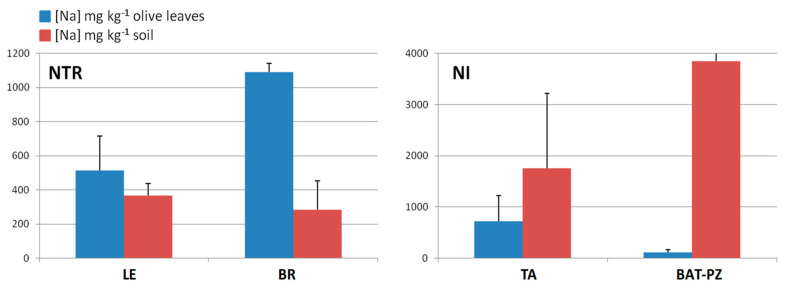
Mean concentration of sodium (Na, mg kg^−1^) within olive tree leaves obtained for infected-not treated (NTR) and not-infected (NI), and the corresponding soil sample sites.

**Figure 6:**
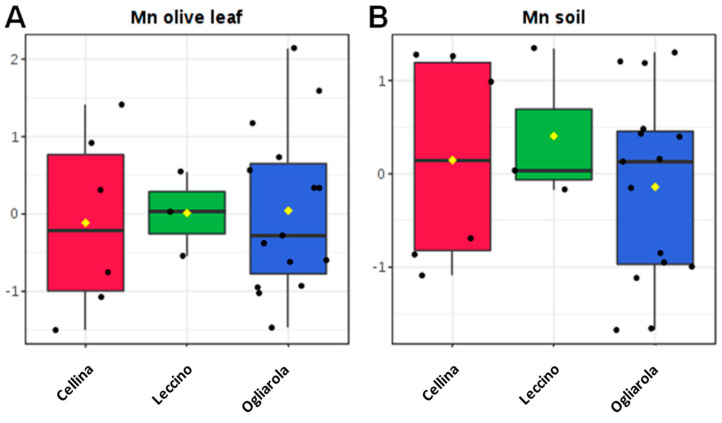
Box plots concentration for manganese olive leaf (**A**) and soil (**B**) samples of infected olive trees of Cellina di Nardò, Leccino and Ogliarola salentina cultivars, located in Taranto province (see also Table 1).

In order to deeply analyze the differences in the macro and micronutrients content, Dentamet^®^-treated (TR) leaf samples obtained from Cellina di Nardò and Ogliarola salentina, all collected in Lecce municipalities, were also compared with all not-infected samples (NI) obtained from BR and TA, as well as from the BAT and PZ provinces. By both unsupervised and supervised multivariate analyses, a good class separation and high predictive ability was observed (R^2^X = 0.48, R^2^Y = 0.82, Q^2^ = 0.77 for OPLS-DA model, Figure 4). As expected, a high relative level of Zn was found for TR with respect to NI samples. Moreover, among the NI olive leaf samples, those collected in Andria, Barletta and Canosa (BAT) as well in Gaudiano (PZ) showed a very high Cu (values ranging from 68.49 to 137.70 mg kg^−1^) and Zn content (values ranging from 37.73 to 56.69 mg kg^−1^). The Mn content was also in the range of normality [28] (i.e., Canosa), with the highest values found in Barletta (i.e., 915 – 1.055 mg kg^−1^). A comparison of Na content in soil with respect to leaf samples taken from not-infected and infected-not treated olive groves is reported in Figure 5. Noteworthily, for the infected trees of Lecce and Brindisi provinces, a remarkably higher Na content was found in the leaves with respect to the corresponding value in the soil. By contrast, an opposite trend was observed for the not-infected trees of Taranto and BAT provinces with a higher Na content in soil with respect to the leaves (Figure 5). We also analyzed the Mn content of soil and leaves of infected Ogliarola salentina, Cellina di Nardò and Leccino trees of Taranto province (Table 1). The Leccino trees showed a relatively higher Mn content in the leaves with respect to the other two cultivars (Figure 6). Real-time PCR showed that *X. f*. subsp. *pauca* was present in all leaf samples taken from farms of the municipalities located in the provinces of Lecce and Brindisi (i.e., the “infected” area), whereas it was not present in the farms located in Taranto province. All of these farms are in the “infected” area. In addition, negative detection was observed for the samples taken from the municipalities of the Barletta-Andria-Trani and Potenza provinces (i.e., the “free” area) (Table 3).

## 3. Discussion

Upon the initial outbreak of OQDS in Salento, recorded during 2008–2009 [13], apparently a single clone of *X. f.* subsp. *pauca*, namely ST53, has been found in the whole Salento area [7]. The typical characteristic of the Salento olive agro-ecosystem, spanning as a *continuum* over many kilometers, most probably favored the further spread of the pathogen in the whole area [16]. The occurrence in this area of two pathogen-sensitive cultivars such as Ogliarola salentina and Cellina di Nardò has represented another key-point for the expansion of the epidemic. Clonal pathogens are favored by large-scale uniformity of environment, so that, upon their adaptation to the new niche, they can flourish in the agro-ecosystem [37]. For plant diseases, the mineral nutrients supplied by soil to the plant in inorganic or organic forms, can also play a role in determining the severity and the spread of the disease [38]. This study highlighted a significant difference in the soil and leaf ionome composition for samples from the Salento peninsula (i.e., LE, BR and TA) with respect to the northern areas (BAT and PZ) where the OQDS has not been found. The unsupervised exploratory (PCA) and supervised classification (PLS-DA, OPLS-DA) data analyses applied in this study revealed a clear separation between the two areas, pointing to a higher Zn, Cu and Mn content found in the northern area as the soil and leaf ionomic profile-discriminating ions. These elements are plant micronutrients essential for plant growth: Zn is involved as cofactor in many enzymes such as alcohol dehydrogenase, carbonic anydrase and RNA polymerase [39]; Cu is essential for the formation of chlorophyll [40], and Mn is involved in the photosynthetic machinery and in the detoxyphication of ROS [41].

It is worth noting that these ions are also deeply involved in the *X. fastidiosa* virulence and in the plant defense systems. Zinc is deeply involved in the mechanisms of plant defense towards pathogens [42]. A zinc-finger protein gene, *CAZFP1*, encodes a zinc-finger transcription factor that is accumulated in the early phase of the infection of *Xanthomonas campetsris* pv. *vesicatoria* to pepper fruits [43]. In addition, zinc fingers binding domains are related to the effector-triggered immune response [44]. High zinc concentrations can protect plants by direct toxicity and by Zn-triggered organic defenses [45,46].

*X. fastidiosa* biofilm formation is inhibited by a copper and zinc concentrations higher than 200 µM and 0.25 mM, respectively [18], and in planta zinc detoxification is required to trigger the full virulence of the pathogen [20]. Within this context, we have shown that the supply to the olive canopy of a zinc–copper–citric acid biocomplex, namely Dentamet^®^, reduced both the field symptoms and *X. f.* subsp. *pauca* cell densities within the foliage enabling the trees to survive to the infection [25]. Recently, a high Mn leaf content would seem to be correlated to a relative degree of tolerance in Leccino cultivar to *X. f.* subsp. *pauca* [22], and the present study would corroborate this feature since both Ogliarola salentina and Cellina di Nardò cultivars are characterized by a lower Mn content than Leccino. This ion is involved in lignin and flavonoid productions that confer protection to the cultivar towards *X. f.* subsp. *pauca* infection [47]. The high content of Cu found in northern areas could be related to the repeated utilization of compounds aiming at controlling phytopathogenic fungi and bacteria such as *Spilocea oleaginea* and *Pseudomonas savastanoi* pv. *savastanoi* commonly found in that area. Meanwhile, the high content of Zn and Mn both in soil and leaves is a feature that deserves further investigations. Olive groves of northern areas (BAT and PZ) also showed a significant high soil content of Na. On the other hand, this ion is not present at a high concentration in the leaves of the cultivar Coratina typical of northern areas. Interestingly, despite the climatic suitability for the survival of *X. f.* subsp. *pauca* [48], there are no outbreaks or record of the pathogen in the northern areas of Apulia, so far. This is in sharp contrast with the expected arrival of the vector in that area from the south, during the last decade at least. Indeed, infection should have already reached north Apulia, taking into account the initial outbreaks [49], the estimated spread of the vector (i.e., 20 km per year) [14] and the possibility for *p. spumarius* to also propagate as a vehicles hitchhiker (EFSA, 2018) [1]. The edaphic properties of the soils and/or a relative tolerance of the local cultivar Coratina, due to a different ionome composition, could partly explain the current situation and deserve further study. The not-infected leaves sampled in olive groves at either the TA or BAT and PZ provinces displayed a low Na content with respect to the infected trees. Moreover, these latter also showed a relevant Na accumulation. This finding confirms what observed in peach trees with symptoms of “phony peach” caused by *X. fastidiosa* [50], and for tobacco leaves infected by *X. fastidiosa* subsp. *fastidiosa* [19]. The accumulation of Na within the infected leaves could be in relation with the leaf wilting incited by the presence of the bacterium within the xylem that causes an increase in solutes and ions to adjust the osmotic potential in that tissue [22]. It has been also observed that the not-infected trees showed a higher Ca content when compared to both the treated and infected trees. This would seem to be in contrast with previous observations that indicate an accumulation of Ca in the xylem of infected grapes, blueberry and pecan [51,52]. However, it should be said that the soils which we studied where non-infected olive trees are growing (i.e., TA, BAT and PZ) are very rich in Ca content, and that the uptake of this ion occurs via the root system, usually through a favorable potential up to the xylem network [53]. In addition, olive cultivars would seem to show a different response in the regulation of Ca-related genes. Ogliarola salentina appears more sensitive than Leccino in showing Ca-induced metabolic changes [22]. This study confirmed and enlarged the observation about the very low Cu content in the leaves found in the infected trees with additional data [11]. In addition to *X. fastidiosa* [19], this finding was also observed in disease caused by other phytopathogenic bacteria, such as *Xanthomonas oryzae* pv. *oryzae* [54] and *Erwinia amylovora* [55]. We have also observed that leaf samples taken from trees that have received the zinc–copper–citric acid biocomplex treatment for two or three years could be significantly discriminated from the infected non treated olive trees growing in the same area, mainly through the Zn, Cu and Na content. A higher content of Zn and Cu is conceivable for the repeated spray treatments to the canopy with the biocomplex, whereas the low Na content could be in relation with metabolic pathways of the trees being close to the normality. It was recently shown that the biocomplex induces an early re-programming of the infected trees upon the spray treatments characterized by the loss of all disease biomarkers [27]. On the other hand, the leaf ionome comparison between treated and not infected trees, as well as between the olive groves of the “free” and “infected” areas also show clear differences, resulting in Zn being the most discriminative ion between the two situations and could be used as a putative biomarker for tolerance to *X. f.* subsp. *pauca*. These findings confirm once more that Dentamet^®^ incites differences in the concentration of the single ions found in the leaves that have received the treatments. [25]. Nevertheless, further work is needed to buttress the present data. In particular, the behavior of Coratina, the most widely planted cultivar north of Salento, would require additional investigation in relation to its susceptibility/tolerance/resistance to *X. f.* subsp. *pauca* infection. This cultivar, indeed, showed a leaf ionome content different from “Ogliarola salentina” and “Cellina di Nardò” cultivars that could be in relation to the progression of the pathogen within the tree.

## 4. Materials and Methods

### 4.1. Soil and Leaf Sampling

**Figure 7:**
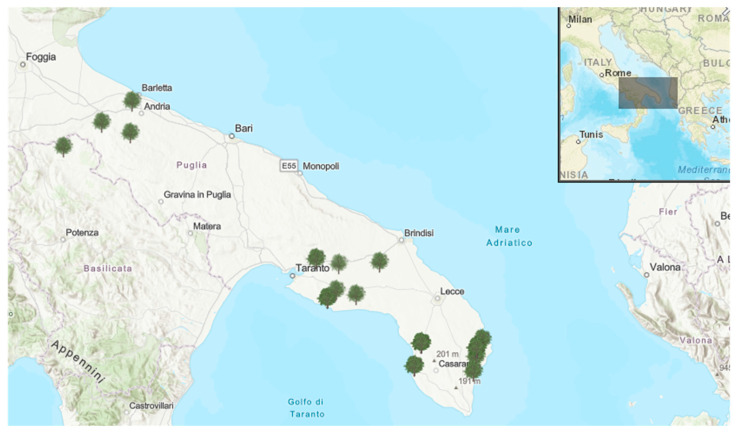
Expansion of Apulia region in Italy. Sample areas are showed by the tree symbol.

In order to verify the ionomic profiles of soils and their availability to olive trees, soil and leaf samples were collected in July 2019 and analyzed. The samples were obtained from the municipalities showed in Table 3 and Figure 7). Annual precipitation (cumulative rainfall) and temperature data for the studied area were reported in Table S4. addition to municipalities of the “infected” area where *X. f*. subsp. *pauca* has been reported, we have also sampled some sites of the not infected area (i.e., BAT and PZ) as well as sites of the “infected” and “containment” areas apparently free from the pathogen (i.e., areas where the official monitoring of the Phytosanitary Service of Apulia Region and further laboratory analyses did not record the bacterium within the municipality territory). We have also assessed some olive farms in the “infected” area that applied a zinc–copper–citric acid biocomplex, namely Dentamet^®^, to control *X. f.* subsp. *pauca* in recent years [25]. The agronomical techniques applied to the olive groves located in the “infected” and “free” areas are as follows: “infected” area, cultivars “Ogliarola salentina” and “Cellina di Nardò”, free vase training system, ample space between the trees, not regular pruning and soil plowing, no irrigation and soil fertilization, control of main pest and pathogens through application of synthetic compounds; “free” area: cultivar “Coratina”, polyconic vase training system, regular annual pruning, plowing, soil fertilization and control of main pests and pathogens through application of synthetic compounds.

From each olive grove, leaves were collected in summer (i.e., July), according to the procedures described by Scortichini et al. [23]. The trees were pruned the year before the sampling and leaf samples were taken from part of the crown of each tree not showing any visible symptom of OQDS. In the cases of infected and treated farms, olive trees showing some visible symptom putatively attributable to OQDS (i.e., leaf scorching, twig and branch dieback) were sampled for confirming the occurrence of the pathogen through real-time PCR assessment by taking asymptomatic leaves [25]. In addition, we performed such an assessment also in the cases of not infected trees that grow in the “infected” area as well as from trees of the “free” area. The leaves sampled and used for the measurements had a healthy appearance, even for the infected and untreated plants. The average water content was always equal to 50% w/w, both for healthy plants and for infected plants. The following elements were analyzed in soil and leaf tissue: calcium (Ca), magnesium (Mg), sodium (Na), iron (Fe), zinc (Zn), copper (Cu), boron (B), manganese (Mn), and molybdenum (Mo). Soil pH was measured in bi-distilled water using a suspension of 1:5 solid to liquid phase. For each farm, three soil and leaf samples were taken according to the methods described elsewhere [23].

### 4.2. Soil and Leaf Analyses

Leaves were washed with distilled water to remove all soil particles and then dried. Each soil (1 g) and leaf (0.5 g) subsample was analyzed separately at the University of Salento using Inductively Coupled Plasma Atomic Emission Spectroscopy (ICP-AES), by following the standard procedures [23,25]. Briefly, samples of known dry weight were mixed with 4 mL H_2_O_2_ and 6 mL HNO_3_ at 180 °C for 10 min, using a microwave digestion system (Milestone Start D). Then they were cooled, diluted with ultrapure water to a final volume of 20 mL, filtered through Whatman No. 42 filter papers, and measured for elemental content using an inductively coupled plasma atomic emission spectrometer (ThermoScientific iCap 6000 Series). Results were expressed as mg·kg^−1^ dry weight.

### 4.3. Statistical and Principal Component Analyses

Standard analysis of variance (One Way-ANOVA) with Tukey’s Honestly Significant Difference (HSD) post hoc test was applied to compare the means between the two cultivation sites and for multiple comparisons of groups using the R statistical environment, Version 3.6.2 on a 64 bit Windows platform (R Development Core Team, 2017) [56]. Moreover, a correlation matrix based on Pearson’s coefficient was calculated for all the measured elements using MetaboAnalyst 4.0, a web-based tool for visualization of metabolomics [57,58]. This approach was used to assess possible linear associations between the variables (Ca, Na, Mg, Fe, Zn, Cu, B, Mn and Mo) of soil data. Multivariate statistical analyses and graphics were obtained using MetaboAnalyst 4.0 software [57,58,59]. Autoscaling pretreatment of the data was carried out to the data prior the analyses [60,61]. Exploratory and classification data analyses were carried out using Principal Component Analysis (PCA), Projection to Latent Structures Discriminant Analysis (PLS-DA) and Orthogonal Projection to Latent Structures Discriminant Analysis (OPLS-DA), both for soil and leaf data set analyses. The statistical models were validated using an internal cross-validation default method (10-fold CV) and evaluated by permutation test statistics. The quality of the models was described by classification accuracy, R^2^ and Q^2^ parameters. The R^2^ value is a cross validation parameter defined as the proportion of variance in the data explained by the models, while the Q^2^ parameter is an internal cross validation parameter, which indicates the predictability of the model. Moreover, the variable contribution was evaluated in the classification models (PLS-DA models), by the VIP score [50] and the box plots for discriminant variables with VIP score > 1 were also evaluated.

### 4.4. Occurrence of Xylella fastidiosa in Olive Groves

The occurrence of *X. fastidiosa* in the olive tree leaves sampled in the “infected” and “free” areas was assessed using real-time PCR [62] by following the procedures described by Scortichini [25]. For these analyses, sampled leaves of the visibly-infected trees of the “infected” area were taken from branches not showing disease symptoms (i.e., leaf wilting or twig dieback). For farms of the “infected” area not showing apparent signs of decline as well as for the farms of the “free” area, the leaf samples were taken from four different twigs representing the four cardinal points.

## Figures and Tables

**Table 1:** Means (mg kg^−1^) ± standard error of the mean (SEM) obtained for the ionomic content of soil samples from different olive farm. Tukey Honestly Significant Difference (HSD) test was applied for multiple comparisons of groups (provinces). BAT: Barletta-Andria-Trani; LE, BR, TA: Lecce, Brindisi, Taranto provinces (Apulia); PZ Potenza province (Basilicata, Apulia bordering region).

	BAT-PZ	BR	LE	TA
B	19.99 ± 0.80	16.90 ± 0.71	17.36 ± 0.87	20.01 ± 1.39
Ca	44.84 × 10^3^ ± 6.93 × 10^3^	31.46 × 10^3^ ± 18.77 × 10^3^	68.21 × 10^3^ ± 6.62 × 10^3^	80.46 × 10^3^ ± 12.19 × 10^3^
Cu	84.24 ± 9.30 ^b^	17.17 ± 4.93 ^a^	30.78 ± 2.38 ^a^	20.27 ± 1.84 ^a^
Fe	20.76 × 10^3^ ± 0.98 × 10^3^	18.88 × 10^3^ ± 0.70 × 10^3^	20.22 × 10^3^ ± 1.03 × 10^3^	19.29 × 10^3^ ± 14.16 × 10^3^
Mg	3.73 × 10^3^ ± 0.14 × 10^3 b^	2.11 × 10^3^ ± 0.36 × 10^3 a^	3.21 × 10^3^ ± 0.11 × 10^3 b^	3.45 × 10^3^ ± 0.14 × 10^3 b^
Mn	0.78 × 10^3^ ± 0.05 × 10^3 ab^	0.60 × 10^3^ ± 0.08 × 10^3 ab^	0.46 × 10^3^ ± 0.06 × 10^3 a^	0.74 × 10^3^ ± 0.079 × 10^3 b^
Mo	0.08 ± 0.02	0.24 ± 0.07	0.27 ± 0.04	0.15 ± 0.03
Na	3.85 × 10^3^ ± 0.02 × 10^3c^	0.27 × 10^3^ ± 0.03 × 10^3 a^	0.62 × 10^3^ ± 0.090 × 10^3 a^	1.75 × 10^3^ ± 0.24 × 10^3 b^
Zn	46.08 ± 1.74 ^a^	24.72 ± 1.16 ^ab^	32.69 ± 2.10 ^b^	34.71 ± 1.92 ^ab^
pH	8.12 ± 0.04	7.64 ± 0.26	7.84 ± 0.11	8.20 ± 0.04

Letters (a, b, c) indicate significant differences for Tukey HSD test at least for 5% statistical probability (see also Supplementary Information Tables S1 and S3). BAT: province of Barletta-Andria-Trani; PZ: province of Potenza (see Table S1 in SI for details); BR: province of Brindisi; LE: province of Lecce; TA: province of Taranto.

**Table 2:** Pearson correlation matrix among the variables for soil samples. *, **, *** indicate significance at *p* < 0.05, *p* < 0.01 and *p* < 0.001, respectively.

	Ca	Cu	Fe	Mg	Mn	Mo	Zn	Na
B	−0.45 ***	0.13	0.89 ***	0.39 ***	0.56 ***	0.02	0.52 ***	0.15
Ca		−0.24 **	−0.59 ***	0.31 ***	−0.37 ***	−0.06	−0.22	−0.03
Cu			0.18 *	0.09	0.16	0.14	0.38 ***	0.23 **
Fe				0.32 ***	0.49 ***	0.15	0.48 ***	0.04
Mg					0.22 *	0.03	0.38 ***	0.19
Mn						−0.19 *	0.46 ***	0.22 **
Mo							0.03	−0.31 **
Zn								0.15

**Table 3:** List of municipalities with the corresponding infection status of the olive farm, cultivar name and number of soil and leaf samples taken for the study. BAT: province of Barletta-Andria-Trani; PZ: province of Potenza; BR: province of Brindisi; LE: province of Lecce; TA: province of Taranto. TR (farm treated with zinc–copper–citric acid), NTR (farm not treated and infected by *Xylella fastidiosa* subsp. *pauca*) or NI (farm not infected by *Xylella fastidiosa* subsp. *pauca*). Ogliarola: Ogliarola salentina; Cellina: Cellina di Nardò. The olive groves of BAT and PZ provinces are located in the “free” area, whereas all the other are located in the “infected” area.

Municipality	Province	Status-Cultivar (*n*° Samples)
Andria	BAT	NI Coratina (3)
Barletta	BAT	NI Coratina (3)
Canosa	BAT	NI Coratina (3)
Gaudiano	PZ	NI Coratina (3)
Francavilla Fontana	BR	NTR Ogliarola (3)
Mesagne	BR	NTR Ogliarola (3)
Galatone	LE	TR Ogliarola (1)/Cellina (2)-NTR Ogliarola (18)
Cannole	LE	TR Cellina (3)-NTR Ogliarola (3)
Giurdignano	LE	TR Ogliarola (3)-NTR Ogliarola (3)/Cellina (3)
Otranto	LE	TR Ogliarola (3)-NTR Cellina (3)
Carpignano	LE	TR Ogliarola (2)/Cellina (1)
Ortelle	LE	NTR Cellina (3)- NTR Ogliarola (3)
Minervino di Lecce	LE	NTR Cellina (3)- NTR Ogliarola (3)
Cursi	LE	NTR Ogliarola (3)
Gallipoli	LE	NTR Ogliarola (24)
Sava	TA	NI Ogliarola (2)/Leccino (1)
Maruggio	TA	NI Ogliarola(1)/Cellina(1)/Leccino(1)
Manduria	TA	NI Ogliarola (1)/Cellina (2)
Torricella	TA	NI Ogliarola (2)/Leccino (1)
Grottaglie	TA	NI Ogliarola (3)/Cellina (3)
Lizzano	TA	NI Ogliarola (6)

## Supplementary Materials

The following are available online at https://www.mdpi.com/2223-7747/9/6/760/s1, Table S1. Standard analysis of variance (One Way-ANOVA) with Tukey’s honestly significant difference (HSD) post hoc test for soil data; Table S2. Means (mg kg^−1^) ± standard error of the mean (SEM) obtained for the ionomic content of soil samples from BAT (province of Barletta-Andria-Trani) and PZ (province of Potenza); Table S3. One-way ANOVA (*p* < 0.05) with the post-hoc test, which includes *p-value* adjustment for multiple comparisons, was applied for the ionomic content of leaf samples from different olive farm. A total of six significant features were found from Tukey Honestly Significant Difference (HSD) test was applied for multiple comparisons of groups (TR, treated with zinc–copper–citric acid, NTR, not treated and infected by *Xylella fastidiosa* subsp. *pauca,* NI, not infected by *Xylella fastidiosa* subsp. *pauca*); Table S4. Annual precipitation (mm of cumulative rainfall) and temperature (°C) data for the studied area Figure S1. Expansion of Apulia region in southern Italy. Variations in Na content (mg kg^−1^) for the considered sample soil sites appear as more or less red, according to four levels (from red: high content, to light yellow: low Na content); Figure S2. Pearson correlation matrix among the variables for soil samples; Figure S3. PCA model performed using the whole data for the Dentamet^®^-treated (TR), not treated-infected (NTR), and not infected (NI) olive leaf samples.
