# Supplementary material for: Soil and Leaf Ionome Heterogeneity in Xylella fastidiosa Subsp. Pauca-Infected, Non-Infected and Treated Olive Groves in Apulia, Italy
Source: Plants (Basel). 2020 Jun 17;9(6):760. doi: 10.3390/plants9060760 (PMC7356509; doi:10.3390/plants9060760)
Supplement: Supplementary file 1 [file plants-09-00760-s001.pdf]

Supplementary Information

Soil and leaf ionome heterogeneity in *Xylella fastidiosa* subsp. *pauca*-infected, non infected and treated olive groves of Apulia, Italy

Laura Del Coco [1], Danilo Migoni [1], Chiara Roberta Girelli [1], Federica Angilè [1], Marco Scortichini [2] and Francesco Paolo Fanizzi [1,3]*

[1] Affiliation 1; e-mail@e-mail.com Department of Biological and Environmental Sciences and Technologies, University of Salento, Prov.le Lecce-Monteroni, I-73100 Lecce, Italy, laura.delcoco@unisalento.it (L.D.C.); danilo.migoni@unisalento.it (D.M.); chiara.girelli@unisalento.it (C.R.G.); federica.angile@unisalento.it (F.A.); fp.fanizzi@unisalento.it (F.P.F.)
[2] Council for Agricultural Research and Agricultural Economy-Research Centre for Olive, Fruit Trees and Citrus, Via di Fioranello, 52, I-00134 Roma, Italy; marco.scortichini@crea.gov.it (M.S.)
[3] University of Salento Local Unit of Consorzio Interuniversitario di Ricerca in Chimica dei Metalli nei Sistemi Biologici (CIRCMSB), Via Celso Ulpiani, 27– 70126 BARI, Italy
* Correspondence: fp.fanizzi@unisalento.it (F.P. Fanizzi)

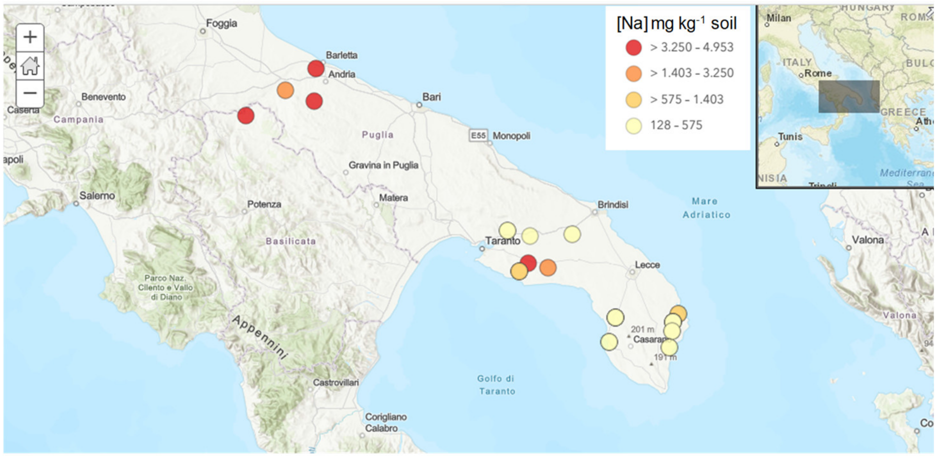

Figure S1. Expansion of Apulia region in southern Italy. Variations in Na content (mg kg^{-1}) for the considered sample soil sites appear as more or less red, according to four levels (from red: high content, to light yellow: low Na content).

Table S1. Standard analysis of variance (One Way-ANOVA) with Tukey's honestly significant difference (HSD) post hoc test for soil data.

```
> AnovaModel.1 <- aov(X.B..ppm ~ province, data=suoli)

> summary(AnovaModel.1)
             Df Sum Sq Mean Sq F value Pr(>F)
province      3    180   59.84   1.218  0.306
Residuals   114   5600   49.12

> with(suoli, numSummary(X.B..ppm, groups=province, statistics=c("mean",
+   "sd")))
           mean       sd data:n
BAT-PT 19.99323 2.775935     12
```

```
BR     16.90092 1.581641      5
LE     17.36196 7.635356     77
TA     20.01121 6.833784     24

> local({
+   .Pairs <- glht(AnovaModel.1, linfct = mcp(province = "Tukey"))
+   print(summary(.Pairs)) # pairwise tests
+   print(confint(.Pairs)) # confidence intervals
+   print(cld(.Pairs)) # compact letter display
+   old.oma <- par(oma=c(0,5,0,0))
+   plot(confint(.Pairs))
+   par(old.oma)
+ })

        Simultaneous Tests for General Linear Hypotheses

Multiple Comparisons of Means: Tukey Contrasts


Fit: aov(formula = X.B..ppm ~ province, data = suoli)

Linear Hypotheses:
                 Estimate Std. Error t value Pr(>|t|)
BR - BAT-PT == 0 -3.09231    3.73056  -0.829    0.830
LE - BAT-PT == 0 -2.63127    2.17513  -1.210    0.604
TA - BAT-PT == 0  0.01798    2.47788   0.007    1.000
LE - BR == 0      0.46104    3.23446   0.143    0.999
TA - BR == 0      3.11029    3.44535   0.903    0.791
TA - LE == 0      2.64925    1.63846   1.617    0.354
(Adjusted p values reported -- single-step method)


        Simultaneous Confidence Intervals

Multiple Comparisons of Means: Tukey Contrasts


Fit: aov(formula = X.B..ppm ~ province, data = suoli)

Quantile = 2.5695
95% family-wise confidence level


Linear Hypotheses:
                 Estimate  lwr       upr
BR - BAT-PT == 0  -3.09231 -12.67789   6.49326
LE - BAT-PT == 0  -2.63127  -8.22020   2.95765
TA - BAT-PT == 0   0.01798  -6.34887   6.38482
LE - BR == 0       0.46104  -7.84981   8.77190
TA - BR == 0       3.11029  -5.74245  11.96303
TA - LE == 0       2.64925  -1.56072   6.85922

BAT-PT     BR     LE     TA
   "a"    "a"    "a"    "a"

> AnovaModel.2 <- aov(X.Ca..ppm ~ province, data=suoli)

> summary(AnovaModel.2)
             Df       Sum Sq    Mean Sq F value Pr(>F)
province      3  16666182329 5555394110   1.797  0.152
Residuals   114 352502785148 3092129694

> with(suoli, numSummary(X.Ca..ppm, groups=province, statistics=c("mean",
+   "sd")))
           mean       sd data:n
BAT-PT 44840.98 24020.56     12
BR     31461.09 41979.51      5
LE     68216.89 58146.51     77
TA     80464.30 59763.86     24
```

```
> local({
+   .Pairs <- glht(AnovaModel.2, linfct = mcp(province = "Tukey"))
+   print(summary(.Pairs)) # pairwise tests
+   print(confint(.Pairs)) # confidence intervals
+   print(cld(.Pairs)) # compact letter display
+   old.oma <- par(oma=c(0,5,0,0))
+   plot(confint(.Pairs))
+   par(old.oma)
+ })

	 Simultaneous Tests for General Linear Hypotheses

Multiple Comparisons of Means: Tukey Contrasts


Fit: aov(formula = X.Ca..ppm ~ province, data = suoli)

Linear Hypotheses:
                 Estimate Std. Error t value Pr(>|t|)
BR - BAT-PT == 0   -13380      29599  -0.452    0.967
LE - BAT-PT == 0    23376      17258   1.355    0.511
TA - BAT-PT == 0    35623      19660   1.812    0.257
LE - BR == 0        36756      25663   1.432    0.462
TA - BR == 0        49003      27336   1.793    0.265
TA - LE == 0        12247      13000   0.942    0.769
(Adjusted p values reported -- single-step method)


	 Simultaneous Confidence Intervals

Multiple Comparisons of Means: Tukey Contrasts


Fit: aov(formula = X.Ca..ppm ~ province, data = suoli)

Quantile = 2.5696
95% family-wise confidence level


Linear Hypotheses:
                 Estimate    lwr         upr        
BR - BAT-PT == 0 -13379.8893 -89438.8520  62679.0733
LE - BAT-PT == 0  23375.9023 -20970.7234  67722.5280
TA - BAT-PT == 0  35623.3133 -14895.8988  86142.5255
LE - BR == 0      36755.7916 -29188.6391 102700.2224
TA - BR == 0      49003.2027 -21240.9112 119247.3165
TA - LE == 0      12247.4110 -21157.5728  45652.3949

BAT-PT     BR     LE     TA 
   "a"    "a"    "a"    "a" 

> AnovaModel.3 <- aov(X.Cu..ppm ~ province, data=suoli)

> summary(AnovaModel.3)
             Df Sum Sq Mean Sq F value   Pr(>F)    
province      3  36993   12331   29.94 2.38e-14 ***
Residuals   114  46951     412                     
---
Signif. codes:  0 '***' 0.001 '**' 0.01 '*' 0.05 '.' 0.1 ' ' 1

> with(suoli, numSummary(X.Cu..ppm, groups=province, statistics=c("mean", 
+   "sd")))
           mean        sd data:n
BAT-PT 84.23687 32.233375     12
BR     17.16790 11.018851      5
LE     30.77773 20.887893     77
TA     20.27396  9.034684     24

> local({
+   .Pairs <- glht(AnovaModel.3, linfct = mcp(province = "Tukey"))
```

```
+   print(summary(.Pairs)) # pairwise tests
+   print(confint(.Pairs)) # confidence intervals
+   print(cld(.Pairs)) # compact letter display
+   old.oma <- par(oma=c(0,5,0,0))
+   plot(confint(.Pairs))
+   par(old.oma)
+ })

        Simultaneous Tests for General Linear Hypotheses

Multiple Comparisons of Means: Tukey Contrasts


Fit: aov(formula = X.Cu..ppm ~ province, data = suoli)

Linear Hypotheses:
                 Estimate Std. Error t value Pr(>|t|)
BR - BAT-PT == 0  -67.069     10.802  -6.209  <0.0001 ***
LE - BAT-PT == 0  -53.459      6.298  -8.488  <0.0001 ***
TA - BAT-PT == 0  -63.963      7.175  -8.915  <0.0001 ***
LE - BR == 0       13.610      9.366   1.453    0.449
TA - BR == 0        3.106      9.977   0.311    0.989
TA - LE == 0      -10.504      4.744  -2.214    0.116
---
Signif. codes:  0 '***' 0.001 '**' 0.01 '*' 0.05 '.' 0.1 ' ' 1
(Adjusted p values reported -- single-step method)


        Simultaneous Confidence Intervals

Multiple Comparisons of Means: Tukey Contrasts


Fit: aov(formula = X.Cu..ppm ~ province, data = suoli)

Quantile = 2.5704
95% family-wise confidence level


Linear Hypotheses:
                 Estimate lwr      upr
BR - BAT-PT == 0 -67.0690 -94.8357 -39.3023
LE - BAT-PT == 0 -53.4591 -69.6487 -37.2696
TA - BAT-PT == 0 -63.9629 -82.4059 -45.5200
LE - BR == 0      13.6098 -10.4644  37.6840
TA - BR == 0       3.1061 -22.5378  28.7500
TA - LE == 0     -10.5038 -22.6989   1.6913

BAT-PT     BR     LE     TA
   "b"    "a"    "a"    "a"

> AnovaModel.4 <- aov(X.Fe..ppm ~ province, data=suoli)

> summary(AnovaModel.4)
             Df     Sum Sq  Mean Sq F value Pr(>F)
province      3   29127999  9709333   0.148  0.931
Residuals   114 7492856881 65726815

> with(suoli, numSummary(X.Fe..ppm, groups=province, statistics=c("mean",
+   "sd")))
           mean       sd data:n
BAT-PT 20761.21 3402.586     12
BR     18880.48 1566.952      5
LE     20228.33 9066.962     77
TA     19291.78 6939.920     24

> local({
+   .Pairs <- glht(AnovaModel.4, linfct = mcp(province = "Tukey"))
+   print(summary(.Pairs)) # pairwise tests
+   print(confint(.Pairs)) # confidence intervals
```

```
+   print(cld(.Pairs)) # compact letter display
+   old.oma <- par(oma=c(0,5,0,0))
+   plot(confint(.Pairs))
+   par(old.oma)
+ })

	 Simultaneous Tests for General Linear Hypotheses

Multiple Comparisons of Means: Tukey Contrasts


Fit: aov(formula = X.Fe..ppm ~ province, data = suoli)

Linear Hypotheses:
                 Estimate Std. Error t value Pr(>|t|)
BR - BAT-PT == 0  -1880.7     4315.4  -0.436    0.970
LE - BAT-PT == 0   -532.9     2516.1  -0.212    0.996
TA - BAT-PT == 0  -1469.4     2866.3  -0.513    0.953
LE - BR == 0       1347.8     3741.5   0.360    0.983
TA - BR == 0        411.3     3985.5   0.103    1.000
TA - LE == 0       -936.5     1895.3  -0.494    0.957
(Adjusted p values reported -- single-step method)


	 Simultaneous Confidence Intervals

Multiple Comparisons of Means: Tukey Contrasts


Fit: aov(formula = X.Fe..ppm ~ province, data = suoli)

Quantile = 2.5698
95% family-wise confidence level
 

Linear Hypotheses:
                 Estimate    lwr         upr        
BR - BAT-PT == 0  -1880.7283 -12970.2935   9208.8369
LE - BAT-PT == 0   -532.8790  -6998.7152   5932.9573
TA - BAT-PT == 0  -1469.4250  -8835.2380   5896.3880
LE - BR == 0       1347.8494  -8266.9947  10962.6934
TA - BR == 0        411.3033  -9830.4440  10653.0507
TA - LE == 0       -936.5460  -5807.0666   3933.9746

BAT-PT     BR     LE     TA 
   "a"    "a"    "a"    "a" 

> AnovaModel.5 <- aov(X.Mg..ppm ~ province, data=suoli)

> summary(AnovaModel.5)
             Df   Sum Sq Mean Sq F value Pr(>F)   
province      3 10335200 3445067   4.363  0.006 **
Residuals   114 90010804  789568                  
---
Signif. codes:  0 '***' 0.001 '**' 0.01 '*' 0.05 '.' 0.1 ' ' 1

> with(suoli, numSummary(X.Mg..ppm, groups=province, statistics=c("mean", 
+   "sd")))
           mean       sd data:n
BAT-PT 3739.368 473.8112     12
BR     2117.892 802.7681      5
LE     3215.729 990.3966     77
TA     3455.760 672.9655     24

> local({
+   .Pairs <- glht(AnovaModel.5, linfct = mcp(province = "Tukey"))
+   print(summary(.Pairs)) # pairwise tests
+   print(confint(.Pairs)) # confidence intervals
+   print(cld(.Pairs)) # compact letter display
+   old.oma <- par(oma=c(0,5,0,0))
```

```
+   plot(confint(.Pairs))
+   par(old.oma)
+ })

	 Simultaneous Tests for General Linear Hypotheses

Multiple Comparisons of Means: Tukey Contrasts


Fit: aov(formula = X.Mg..ppm ~ province, data = suoli)

Linear Hypotheses:
                 Estimate Std. Error t value Pr(>|t|)
BR - BAT-PT == 0  -1621.5      473.0  -3.428   0.0041 **
LE - BAT-PT == 0   -523.6      275.8  -1.899   0.2192
TA - BAT-PT == 0   -283.6      314.2  -0.903   0.7911
LE - BR == 0       1097.8      410.1   2.677   0.0379 *
TA - BR == 0       1337.9      436.8   3.063   0.0130 *
TA - LE == 0        240.0      207.7   1.155   0.6385
---
Signif. codes:  0 '***' 0.001 '**' 0.01 '*' 0.05 '.' 0.1 ' ' 1
(Adjusted p values reported -- single-step method)


	 Simultaneous Confidence Intervals

Multiple Comparisons of Means: Tukey Contrasts


Fit: aov(formula = X.Mg..ppm ~ province, data = suoli)

Quantile = 2.5698
95% family-wise confidence level


Linear Hypotheses:
                 Estimate   lwr        upr
BR - BAT-PT == 0 -1621.4763 -2836.9227  -406.0300
LE - BAT-PT == 0  -523.6389 -1232.3120   185.0343
TA - BAT-PT == 0  -283.6083 -1090.9214   523.7047
LE - BR == 0      1097.8375    44.0246  2151.6504
TA - BR == 0      1337.8680   215.3448  2460.3912
TA - LE == 0       240.0305  -293.7917   773.8527

BAT-PT     BR     LE     TA
   "b"    "a"    "b"    "b"

> AnovaModel.6 <- aov(X.Mn..ppm ~ province, data=suoli)

> summary(AnovaModel.6)
             Df   Sum Sq Mean Sq F value Pr(>F)
province      3  2087030  695677   3.499 0.0179 *
Residuals   114 22666453  198829
---
Signif. codes:  0 '***' 0.001 '**' 0.01 '*' 0.05 '.' 0.1 ' ' 1

> with(suoli, numSummary(X.Mn..ppm, groups=province, statistics=c("mean",
+   "sd")))
           mean       sd data:n
BAT-PT 780.4618 174.9657     12
BR     603.7944 178.6448      5
LE     465.0300 496.6594     77
TA     742.5457 387.5841     24

> local({
+   .Pairs <- glht(AnovaModel.6, linfct = mcp(province = "Tukey"))
+   print(summary(.Pairs)) # pairwise tests
+   print(confint(.Pairs)) # confidence intervals
+   print(cld(.Pairs)) # compact letter display
+   old.oma <- par(oma=c(0,5,0,0))
```

```
+   plot(confint(.Pairs))
+   par(old.oma)
+ })

        Simultaneous Tests for General Linear Hypotheses

Multiple Comparisons of Means: Tukey Contrasts


Fit: aov(formula = X.Mn..ppm ~ province, data = suoli)

Linear Hypotheses:
                 Estimate Std. Error t value Pr(>|t|)
BR - BAT-PT == 0  -176.67     237.35  -0.744   0.8705
LE - BAT-PT == 0  -315.43     138.39  -2.279   0.1003
TA - BAT-PT == 0   -37.92     157.65  -0.241   0.9947
LE - BR == 0      -138.76     205.79  -0.674   0.8999
TA - BR == 0       138.75     219.20   0.633   0.9154
TA - LE == 0       277.52     104.24   2.662   0.0396 *
---
Signif. codes:  0 '***' 0.001 '**' 0.01 '*' 0.05 '.' 0.1 ' ' 1
(Adjusted p values reported -- single-step method)


        Simultaneous Confidence Intervals

Multiple Comparisons of Means: Tukey Contrasts


Fit: aov(formula = X.Mn..ppm ~ province, data = suoli)

Quantile = 2.5692
95% family-wise confidence level


Linear Hypotheses:
                 Estimate  lwr       upr
BR - BAT-PT == 0 -176.6674 -786.4598  433.1249
LE - BAT-PT == 0 -315.4318 -670.9749   40.1112
TA - BAT-PT == 0  -37.9161 -442.9470  367.1148
LE - BR == 0     -138.7644 -667.4649  389.9361
TA - BR == 0      138.7513 -424.4213  701.9239
TA - LE == 0      277.5157    9.6958  545.3356

BAT-PT     BR     LE     TA
  "ab"   "ab"    "a"    "b"

> AnovaModel.7 <- aov(X.Mo..ppm ~ province, data=suoli)

> summary(AnovaModel.7)
             Df Sum Sq Mean Sq F value Pr(>F)
province      3  0.556 0.18523    1.98  0.121
Residuals   114 10.664 0.09354

> with(suoli, numSummary(X.Mo..ppm, groups=province, statistics=c("mean",
+   "sd")))
             mean         sd data:n
BAT-PT 0.08486237 0.06712849     12
BR     0.24476198 0.14974666      5
LE     0.27444941 0.36193560     77
TA     0.15098259 0.15725193     24

> local({
+   .Pairs <- glht(AnovaModel.7, linfct = mcp(province = "Tukey"))
+   print(summary(.Pairs)) # pairwise tests
+   print(confint(.Pairs)) # confidence intervals
+   print(cld(.Pairs)) # compact letter display
+   old.oma <- par(oma=c(0,5,0,0))
+   plot(confint(.Pairs))
+   par(old.oma)
```

```
+ })

	 Simultaneous Tests for General Linear Hypotheses

Multiple Comparisons of Means: Tukey Contrasts


Fit: aov(formula = X.Mo..ppm ~ province, data = suoli)

Linear Hypotheses:
                 Estimate Std. Error t value Pr(>|t|)
BR - BAT-PT == 0  0.15990    0.16280   0.982    0.746
LE - BAT-PT == 0  0.18959    0.09492   1.997    0.181
TA - BAT-PT == 0  0.06612    0.10813   0.611    0.923
LE - BR == 0      0.02969    0.14115   0.210    0.996
TA - BR == 0     -0.09378    0.15035  -0.624    0.919
TA - LE == 0     -0.12347    0.07150  -1.727    0.297
(Adjusted p values reported -- single-step method)


	 Simultaneous Confidence Intervals

Multiple Comparisons of Means: Tukey Contrasts


Fit: aov(formula = X.Mo..ppm ~ province, data = suoli)

Quantile = 2.5701
95% family-wise confidence level


Linear Hypotheses:
                 Estimate lwr      upr
BR - BAT-PT == 0  0.15990 -0.25850  0.57830
LE - BAT-PT == 0  0.18959 -0.05437  0.43354
TA - BAT-PT == 0  0.06612 -0.21179  0.34403
LE - BR == 0      0.02969 -0.33308  0.39245
TA - BR == 0     -0.09378 -0.48020  0.29264
TA - LE == 0     -0.12347 -0.30723  0.06030

BAT-PT     BR     LE     TA 
   "a"    "a"    "a"    "a" 

> AnovaModel.8 <- aov(X.Na..ppm ~ province, data=suoli)

> summary(AnovaModel.8)
             Df    Sum Sq  Mean Sq F value Pr(>F)    
province      3 116426154 38808718   49.61 <2e-16 ***
Residuals   100  78231330   782313                   
---
Signif. codes:  0 '***' 0.001 '**' 0.01 '*' 0.05 '.' 0.1 ' ' 1
14 observations deleted due to missingness

> with(suoli, numSummary(X.Na..ppm, groups=province, statistics=c("mean", 
+   "sd")))
            mean         sd data:n data:NA
BAT-PT 3850.0000  842.69503     12       0
BR      274.4000   66.88273      5       0
LE      625.9683  794.80322     63      14
TA     1750.9583 1165.36613     24       0

> local({
+   .Pairs <- glht(AnovaModel.8, linfct = mcp(province = "Tukey"))
+   print(summary(.Pairs)) # pairwise tests
+   print(confint(.Pairs)) # confidence intervals
+   print(cld(.Pairs)) # compact letter display
+   old.oma <- par(oma=c(0,5,0,0))
+   plot(confint(.Pairs))
+   par(old.oma)
+ })
```

```
	 Simultaneous Tests for General Linear Hypotheses

Multiple Comparisons of Means: Tukey Contrasts


Fit: aov(formula = X.Na..ppm ~ province, data = suoli)

Linear Hypotheses:
                 Estimate Std. Error t value Pr(>|t|)    
BR - BAT-PT == 0  -3575.6      470.8  -7.595  < 0.001 ***
LE - BAT-PT == 0  -3224.0      278.6 -11.573  < 0.001 ***
TA - BAT-PT == 0  -2099.0      312.7  -6.712  < 0.001 ***
LE - BR == 0        351.6      411.0   0.856  0.81741    
TA - BR == 0       1476.6      434.8   3.396  0.00493 ** 
TA - LE == 0       1125.0      212.2   5.302  < 0.001 ***
---
Signif. codes:  0 '***' 0.001 '**' 0.01 '*' 0.05 '.' 0.1 ' ' 1
(Adjusted p values reported -- single-step method)


	 Simultaneous Confidence Intervals

Multiple Comparisons of Means: Tukey Contrasts


Fit: aov(formula = X.Na..ppm ~ province, data = suoli)

Quantile = 2.5793
95% family-wise confidence level
 

Linear Hypotheses:
                 Estimate   lwr        upr       
BR - BAT-PT == 0 -3575.6000 -4789.9418 -2361.2582
LE - BAT-PT == 0 -3224.0317 -3942.5898 -2505.4737
TA - BAT-PT == 0 -2099.0417 -2905.6210 -1292.4623
LE - BR == 0       351.5683  -708.3962  1411.5327
TA - BR == 0      1476.5583   355.0552  2598.0614
TA - LE == 0      1124.9901   577.7526  1672.2276

BAT-PT     BR     LE     TA 
   "c"    "a"    "a"    "b" 

> AnovaModel.9 <- aov(X.Zn..ppm ~ province, data=suoli)

> summary(AnovaModel.9)
             Df Sum Sq Mean Sq F value Pr(>F)  
province      3   2324   774.6   3.113 0.0291 *
Residuals   114  28363   248.8                 
---
Signif. codes:  0 '***' 0.001 '**' 0.01 '*' 0.05 '.' 0.1 ' ' 1

> with(suoli, numSummary(X.Zn..ppm, groups=province, statistics=c("mean", 
+   "sd")))
           mean        sd data:n
BAT-PT 46.08055  6.017031     12
BR     24.72074  2.591203      5
LE     32.69199 18.457997     77
TA     34.71142  9.429347     24

> local({
+   .Pairs <- glht(AnovaModel.9, linfct = mcp(province = "Tukey"))
+   print(summary(.Pairs)) # pairwise tests
+   print(confint(.Pairs)) # confidence intervals
+   print(cld(.Pairs)) # compact letter display
+   old.oma <- par(oma=c(0,5,0,0))
+   plot(confint(.Pairs))
+   par(old.oma)
+ })
```

```
        Simultaneous Tests for General Linear Hypotheses

Multiple Comparisons of Means: Tukey Contrasts


Fit: aov(formula = X.Zn..ppm ~ province, data = suoli)

Linear Hypotheses:
                 Estimate Std. Error t value Pr(>|t|)
BR - BAT-PT == 0  -21.360      8.396  -2.544   0.0533 .
LE - BAT-PT == 0  -13.389      4.895  -2.735   0.0330 *
TA - BAT-PT == 0  -11.369      5.577  -2.039   0.1672
LE - BR == 0        7.971      7.279   1.095   0.6768
TA - BR == 0        9.991      7.754   1.288   0.5530
TA - LE == 0        2.019      3.688   0.548   0.9430
---
Signif. codes:  0 '***' 0.001 '**' 0.01 '*' 0.05 '.' 0.1 ' ' 1
(Adjusted p values reported -- single-step method)


        Simultaneous Confidence Intervals

Multiple Comparisons of Means: Tukey Contrasts


Fit: aov(formula = X.Zn..ppm ~ province, data = suoli)

Quantile = 2.5715
95% family-wise confidence level


Linear Hypotheses:
                 Estimate lwr      upr
BR - BAT-PT == 0 -21.3598 -42.9499   0.2303
LE - BAT-PT == 0 -13.3886 -25.9768  -0.8003
TA - BAT-PT == 0 -11.3691 -25.7095   2.9713
LE - BR == 0       7.9712 -10.7478  26.6903
TA - BR == 0       9.9907  -9.9488  29.9302
TA - LE == 0       2.0194  -7.4629  11.5018

BAT-PT     BR     LE     TA
   "a"   "ab"    "b"   "ab"

> AnovaModel.1 <- aov(pH ~ province, data=terreni)

> summary(AnovaModel.1)
             Df Sum Sq Mean Sq F value Pr(>F)
province      3   3.24  1.0784   1.872  0.138
Residuals   119  68.56  0.5761
3 observations deleted due to missingness

> with(terreni, numSummary(pH, groups=province, statistics=c("mean", "sd")))
          mean        sd data:n data:NA
BAT-PT 8.12250 0.1218885     12       0
BR     7.74500 0.5751087      6       0
LE     7.83037 0.9081691     81       3
TA     8.20250 0.1817129     24       0

> local({
+   .Pairs <- glht(AnovaModel.1, linfct = mcp(province = "Tukey"))
+   print(summary(.Pairs)) # pairwise tests
+   print(confint(.Pairs)) # confidence intervals
+   print(cld(.Pairs)) # compact letter display
+   old.oma <- par(oma=c(0,5,0,0))
+   plot(confint(.Pairs))
+   par(old.oma)
+ })

        Simultaneous Tests for General Linear Hypotheses
```

```
Multiple Comparisons of Means: Tukey Contrasts


Fit: aov(formula = pH ~ province, data = terreni)

Linear Hypotheses:
                    Estimate Std. Error t value Pr(>|t|)
BR - BAT-PT == 0 -0.37750    0.37951  -0.995    0.739
LE - BAT-PT == 0 -0.29213    0.23478  -1.244    0.583
TA - BAT-PT == 0  0.08000    0.26836   0.298    0.990
LE - BR == 0      0.08537    0.32114   0.266    0.993
TA - BR == 0      0.45750    0.34645   1.321    0.534
TA - LE == 0      0.37213    0.17640   2.110    0.145
(Adjusted p values reported -- single-step method)


        Simultaneous Confidence Intervals

Multiple Comparisons of Means: Tukey Contrasts


Fit: aov(formula = pH ~ province, data = terreni)

Quantile = 2.5715
95% family-wise confidence level


Linear Hypotheses:
                    Estimate lwr      upr
BR - BAT-PT == 0 -0.37750 -1.35342  0.59842
LE - BAT-PT == 0 -0.29213 -0.89587  0.31161
TA - BAT-PT == 0  0.08000 -0.61008  0.77008
LE - BR == 0      0.08537 -0.74045  0.91119
TA - BR == 0      0.45750 -0.43339  1.34839
TA - LE == 0      0.37213 -0.08149  0.82575

BAT-PT     BR      LE      TA
   "a"    "a"     "a"     "a"
```

Table S2. Means (mg kg^{-1}) ± standard error of the mean (SEM) obtained for the ionomic content of soil samples from BAT (province of Barletta-Andria-Trani) and PZ (province of Potenza).

**province**	**BAT**	**PZ**
**B**	19.33±0.20	21.99±0.20
**Ca**	54828.13±6186	14879.53±390.81
**Cu**	97.43±8.47	44.67±0.92
**Fe**	20531.53±1315.84	21450.23±363.24
**Mg**	3677.06±179.02	3926.30±60.76
**Mn**	752.91±65.46	863.11±12.30
**Mo**	0.09±0.03	0.08±0.02
**Zn**	45.71±2.34	47.19±0.28
**Na**	3521.89±232.37	4834.33±89.59
**pH**	8.12±0.05	8.14±0.00

Figure S1. Pearson correlation matrix among the variables for soil samples.

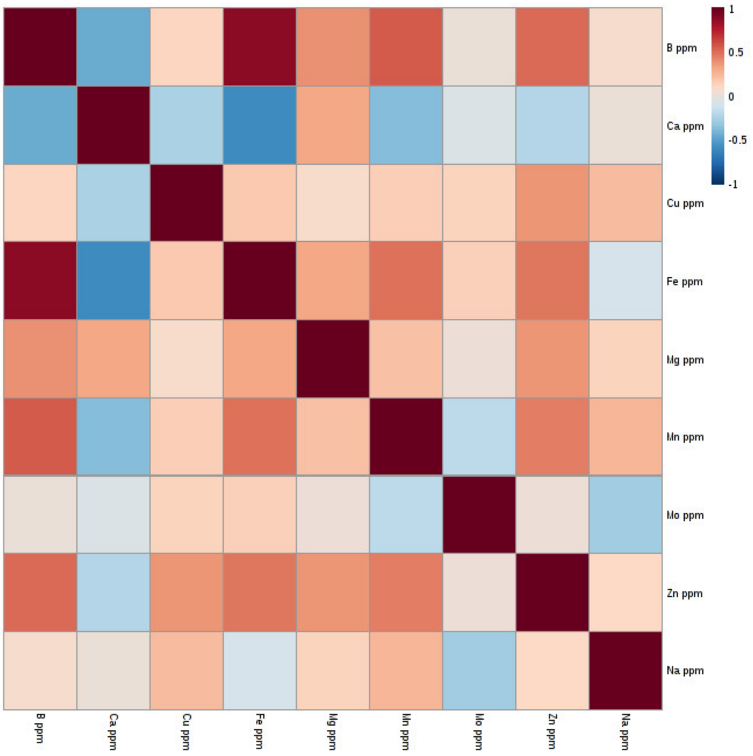

Figure S2. PCA model performed using the whole data for the Dentamet®-treated (TR), not treated-infected (NTR), and not infected (NI) olive leaf samples.

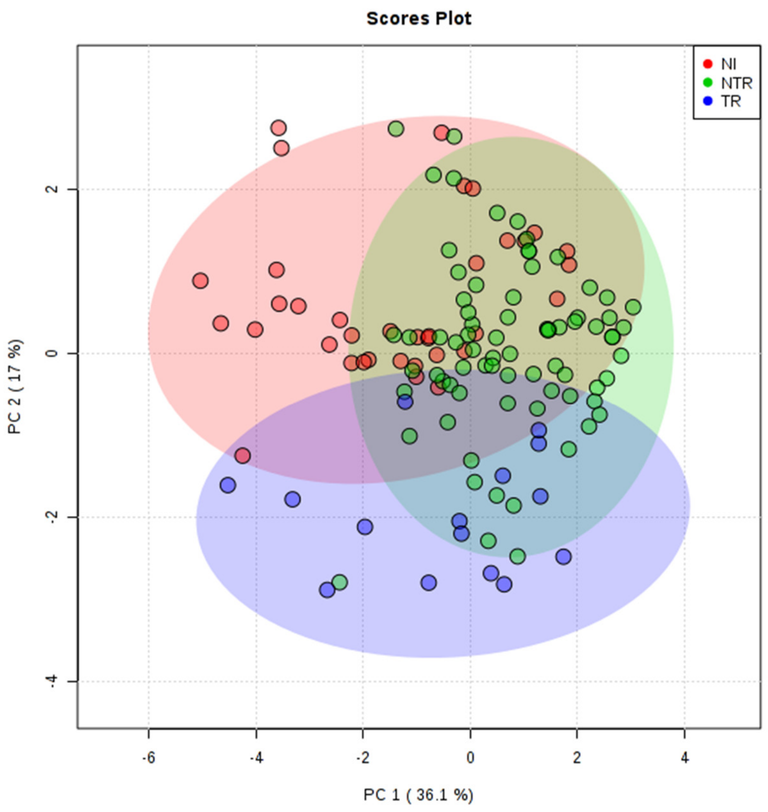

Table S3. One-way ANOVA (p< 0.05) with the post-hoc test, which includes *p-value* adjustment for multiple comparisons, was applied for the ionomic content of leaf samples from different olive farm. A total of six significant features were found from Tukey Honestly Significant Difference (HSD) test was applied for multiple comparisons of groups (TR, treated with zinc-copper-citric acid, NTR, not treated and infected by *Xylella fastidiosa* subsp. *pauca,* NI, not infected by *Xylella fastidiosa* subsp. *pauca*).

id	f.value	p.value	FDR	Tukey's HSD
**Zn**	101.07	1.09E-26	9.79E-26	NTR-NI; TR-NI; TR-NTR
**Ca**	22.105	6.29E-09	2.83E-08	NTR-NI; TR-NI
**Cu**	16.211	5.64E-07	1.69E-06	NTR-NI; TR-NTR
**Mn**	12.185	1.49E-05	3.34E-05	NTR-NI; TR-NI
**Fe**	10.921	4.31E-05	7.75E-05	NTR-NI; TR-NI
**Mo**	9.3243	0.00016967	0.00025451	NTR-NI
**Mg**	6.8693	0.0014854	0.0019098	NTR-NI
**Na**	5.54	0.0049689	0.00559	TR-NI; TR-NTR
**B**	4.1127	0.018667	0.018667	TR-NTR

Table S4. Annual precipitation (mm of cumulative rainfall) and temperature (°C) data for the studied area

**meteorological stations**	**Province**	**Annual cumulative rainfall (millimetres)**	**average annual maximum temperature (T°C)**	**average annual minimum temperature (T°C)**
Barletta	BAT	466.6		
Andria	BAT	697	21.5	12
Castel del Monte	BAT	765.6	20.7	9.3
Bisceglie	BAT	580	21.3	13.1
**provinces average**	**BAT**	**627.3**	**21.16666667**	**11.46666667**
Novoli	LE	662.2	22.2	12.8
Lecce	LE	326.8	22.3	13
Melendugno	LE	721.4	22.2	13.7
Corigliano	LE	867.6		
Maglie	LE	765.4	21.9	13.6
Otranto	LE	898		
Minervino	LE	947.6	22.1	14
Vignacastrisi	LE	1025.2	20.9	12.8
Ruffano	LE	720	21.8	13.3
S.M.diLeuca	LE	747.4	21.2	14.5
Presicce	LE	730.6	22	13.5
Alessano	LE	761.6		
Taviano	LE	488.4	22.3	13.9
Collepasso	LE	618	21.6	13.3
Gallipoli	LE	513.6	22.1	15.2
Galatina	LE	811.4	21.8	13.1
Nardò	LE	641.2	22.3	13.7

**provinces average**	**LE**	**720.3764706**	**21.90714286**	**13.6**
Martina Franca	TA	893.4	19.1	11.7
Mottola	TA	593.6	20.2	12.1
Massafra	TA	666.8	22.1	14.2
Crispiano	TA	577.6	20.9	12.8
Manduria	TA	724.8	22.5	13.7
Lizzano	TA	546.8	23.7	14.8
Grottaglie	TA	820.8	22.2	12.9
S. Giorgio Ionico	TA	530.4	22.9	13.6
Taranto	TA	532	23.3	15.6
Talsano	TA	459.6	20.9	12
**provinces average**	**TA**	**634.58**	**21.78**	**13.34**
Melfi	PZ	823.8	18.9	8.5
Lavello	PZ	505.6	22.1	13.4
Venosa	PZ	652.8	22.1	11.6
Ripacandida	PZ	693.8	19.1	10.5
**provinces average**	**PZ**	**669**	**20.55**	**11**
**https://protezionecivile.puglia.it/centro-funzionale-decentrato/rete-di-monitoraggio/annali-e-dati-idrologici-elaborati/annali-idrologici-parte-i-download/**				
